# Delivery of malaria services during a pandemic: lessons from COVID-19 in Nigeria

**DOI:** 10.1093/inthealth/ihaf074

**Published:** 2025-07-08

**Authors:** Emma K Manning, Olusola Oresanya, James K Tibenderana, Kolawole Maxwell

**Affiliations:** Malaria Consortium, 244-254 Cambridge Heath Road, London E2 9DA, UK; Malaria Consortium, 33 Pope John Paul II St, Maitama 904101, Abuja, Federal Capital Territory, Nigeria; Malaria Consortium, 244-254 Cambridge Heath Road, London E2 9DA, UK; Malaria Consortium, 33 Pope John Paul II St, Maitama 904101, Abuja, Federal Capital Territory, Nigeria

**Keywords:** COVID-19, malaria, Nigeria, pandemic response, seasonal malaria chemoprevention

## Abstract

The coronavirus disease 2019 (COVID-19) pandemic posed significant threats to maintaining malaria services in Nigeria and threatened to reverse global progress towards elimination of the disease. During the COVID-19 pandemic, we worked in collaboration with the National Malaria Elimination Programme in Nigeria across 11 states to ensure that malaria campaigns and routine services continued. Here, we share the challenges and experiences from developing and implementing operational guidelines that enabled programmes to be adapted during unpredictable situations. The modifications made to long-lasting insecticide-treated net distribution and seasonal malaria chemoprevention campaign strategies enabled wide coverage of these interventions, despite limitations imposed by lockdowns. Strong partnerships were essential for the continued delivery of malaria services during lockdowns, which also highlighted the importance of community health workers during emergencies.

## Background

Over the past two decades Nigeria has made great progress to reduce malaria morbidity and mortality. From 2008 to 2019, malaria incidence and mortality declined by 27% and 37%, respectively.^1^ When coronavirus disease 2019 (COVID-19) emerged and governments began to impose restriction of movement, the global malaria community became increasingly concerned about the impact that the disease, and lockdowns, would have on the delivery of malaria services. In March 2020, the WHO issued a statement urging countries to ensure the continuity of malaria services during the pandemic or risk a substantial increase in deaths.^2^ Malaria Consortium has been working in partnership with the Ministry of Health in Nigeria since 2008; when the first COVID-19 case was reported in February 2020, we were supporting in 11 states. In this article, we outline the steps taken to maintain malaria services during the pandemic, and the challenges and enablers of these actions. We hope these lessons will support better preparedness for future threats.

## Initial challenges

### Underestimations and uncertainty

Until the first case of COVID-19 was confirmed in Nigeria, policymakers in the country viewed the disease as a distant threat. This resulted in a hesitancy to impose restrictions on movement for fear of the economic impact. In addition, as Nigeria had previously mounted successful responses to epidemics, such as Ebola, there was a general feeling that the country would be well prepared and able to interrupt transmission quickly. However, unlike previous epidemics the country had handled, the novelty of COVID-19 and the absence of diagnostics, and therefore data, made it difficult for decision makers to assess the threat and determine if a liberal or conservative approach should be taken.

### Urgency and unintended consequences

In March 2020, the Government of Nigeria declared a lockdown across the entire country that restricted movement, limited malaria control activities and had significant economic implications. The countrywide lockdown presented several challenges for the malaria programme including impeding communication between the National Malaria Elimination Programme (NMEP) and partners, delaying procurement of malaria commodities and reducing the flow of data between regions. This impacted the availability of malaria commodities, resulting in stockouts in health facilities and exacerbating existing challenges to malaria service delivery. In addition, despite guidance to remain open, many health facilities closed due to receiving an influx of patients with common illnesses and suspected COVID-19. As health facilities became overwhelmed, access to routine diagnostic and treatment services was severely limited, prompting urgent action to adapt guidelines and delivery methods to the situation.

## Mobilising a response

### Re-establishing communication

The first step to maintaining malaria services was to re-establish communication channels within the NMEP and between the NMEP and external partners. Previously, meetings had been largely in-person, and the restrictions on movement and meetings meant it was difficult to conduct business with a group remotely. To facilitate communication (Zoom Video Communications, Inc. San Jose, California, USA), Zoom licenses were purchased on behalf of the NMEP and state malaria elimination programmes in Lagos and Kano, while partners in other areas purchased licenses for other states. The NMEP began scheduling virtual meetings, first with internal staff and then with partner organisations, which enabled planning and coordination while lockdown restrictions were in place.

### Developing and adapting guidance

In the first virtual meeting, it was established that malaria services would continue despite the lockdowns and that a guidance document was needed to provide information on the adaptations to delivery of essential services to maintain the safety of staff and community members. To support the NMEP to develop guidelines, we drew upon guidance from the WHO, including the WHO's interim operational guidance for maintaining the continuity of essential health services and tailoring malaria interventions during the COVID-19 pandemic together with our own organisational global outbreak management plan in response to COVID-19. The guidance for these documents was then contextualised to align with the Nigerian Centre for Disease Control's response.^3,4^ In addition, we worked with the NMEP to identify vulnerable points in their existing strategies that could lead to community members or field staff being exposed to COVID-19, and created a revised strategy to mitigate these risks. The adapted guidance for delivering malaria campaigns and routine services in Nigeria was then documented in the NMEP Guidelines for Business Continuity in Emergency.^5^

### Delivering campaigns and services under new guidance

#### Maintaining high coverage of long-lasting insecticidal nets

To ensure long-lasting insecticidal nets (LLINs) could be delivered safely, the delivery strategy was adapted from a double-stage fixed-point strategy to a single-phase door-to-door strategy. A double-phase fixed-point strategy involves campaign teams visiting households to raise awareness of the campaign and deliver net cards, then household members travel to a designated place to redeem their net cards and collect nets. By contrast, a single-phase door-to-door strategy involves a single visit where nets are delivered directly to the household at the same time as awareness raising. This strategy reduced the mixing of different households that would usually occur when collecting nets at a fixed location. The door-to-door approach required health workers to carry a large number of nets between households so wheelbarrows were provided to facilitate this. During the campaign, health workers were provided with personal protective equipment (PPE) to reduce the risk of transmission between households and health workers. This distribution approach achieved high coverage of nets; however, this method came with additional costs and required greater effort by health workers.

#### Adapting seasonal malaria chemoprevention delivery

Seasonal malaria chemoprevention (SMC) campaigns were also adapted to reduce the risk of transmission between health workers and community members. Based on WHO guidelines, protocols were developed for implementing SMC during the pandemic that included implementing adequate infection prevention and control measures such as maintaining two metres of distance where possible, wearing PPE and handwashing or sanitising between visits. The method of delivery was also modified; instead of health workers administering the first dose to children, caregivers were shown from a safe distance how to give the medicines to the child. This reduced the need for health workers to enter households or have close contact with community members.

#### Safeguarding case management services

In response to restrictions on movement, and many health facilities becoming overwhelmed and closing, it was clear that communities would increasingly rely on health services provided by community health workers (CHWs) as the closest point of care. Recognising the increased risk to health workers, we supported the National Primary Health Care Development Agency to revise the CHWs’ guide for implementing integrated community case management. This guide included infection prevention and control measures and training for CHWs on how to reduce the risk of COVID-19 transmission while providing health services. CHWs were also trained to deliver information on COVID-19, including how to prevent transmission, disease symptoms and what to do if a case was suspected.

## Lessons and recommendations

In this retrospective review, we reflect on the lessons learnt from supporting the NMEP to maintain malaria services throughout the COVID-19 pandemic. The adaptations made to LLIN campaigns during the COVID-19 pandemic were not only effective for net distribution, but also fostered equity within communities. Distributing LLINs using the door-to-door method eliminated the gap between the number of households mobilised and the number who collected nets from the distribution points. Moreover, when visiting households with the LLINs, CHWs were able to demonstrate how to hang the nets, which improved their proper use. Although direct distribution achieved high coverage, it was more expensive than the typical fixed-point distribution system. In addition, the adaptations made to SMC delivery meant that children were more relaxed when receiving medicines from a family member rather than from a CHW. These adaptations of programming highlight the need for flexible donor funding in emergency situations to enable teams to respond to context-based needs and maintain impact of services.

Digital platforms were an essential component of mobilising a response, enabling meetings with individuals across the world, which meant that additional knowledge and expertise could be shared easily. Despite clear advantages to online meetings during the pandemic, there were some drawbacks. Delivering training sessions virtually was suboptimal as many participants were not familiar with using virtual platforms and trainers could not assess if participants had fully understood the content due to lower levels of active participation. Furthermore, it was difficult to determine participants’ understanding of the training content as individuals could join the virtual meetings without participating and there was limited oversight to confirm their engagement. Strategies are needed to adapt training to suit virtual delivery and validate engagement with the topic to ensure that those who attend training have adequately understood the content.

The experiences presented here offer insights into barriers and facilitators when providing malaria services during periods of instability. The adaptations required to continue malaria services during the pandemic led to the development of effective and agile delivery methods suited to the lockdown conditions. To improve future preparedness, it is essential to invest in digital tools to facilitate data sharing and strengthen coordination platforms to support decision making. Although most operational guidelines have reverted to prepandemic standards, the lessons from COVID-19 must not be forgotten.

## Data Availability

The data underlying this article will be shared upon reasonable request to the corresponding author.
